# Cemento-Ossifying Fibroma in a Patient with End-Stage Renal Disease

**DOI:** 10.1155/2013/923128

**Published:** 2013-05-30

**Authors:** Divya Gopinath, V. T. Beena, G. Sugirtharaj, K. Vidhyadharan, K. Salmanul Faris, Sajai J. Kumar

**Affiliations:** ^1^Department of Oral Pathology & Microbiology, Government of Dental College, Calicut, Kerala 673008, India; ^2^Department of Oral & Maxillofacial Pathology, Government of Dental College, Trivandrum Kerala 695011, India

## Abstract

The presence of chronic renal disease (CRD) is a predisposing factor for the occurrence of soft and hard tissue lesions in the oral cavity. The cemento-ossifying fibroma (COF) is an uncommon benign fibroosseous lesion composed of fibrocellular component and calcified materials like cementum and woven bone. A 37-year-old female patient undergoing chronic haemodialysis reported to our institution with a complaint of slow growing, nontender swelling of mandible of 6-month duration. Computed tomography disclosed an ill-defined lesion showing thinning and expansion of buccal as well as lingual cortical plate with flecks of radiopacity in centre. Incision biopsy revealed histological characteristics consistent with cemento-ossifying fibroma. The lesion was excised under local anesthesia. The histopathological examination revealed irregularly shaped bone and cementum-like hard tissue calcifications contained within hypercellular fibrous tissue stroma, leading to a confirmation of the diagnosis of cemento-ossifying fibroma. This paper aims to provide light to the fact that the soft and hard tissues of the oral region may become susceptible to the development of pathological growths in case of some particular systemic conditions.

## 1. Introduction

Chronic renal disease (CRD) can be defined as the progressive and irreversible decline in kidney functions and up to 90% of patients having renal insufficiency may present with oral signs and symptoms in soft and hard tissues either as a result of the disease itself or the treatment protocol [1]. Halitosis, altered taste and sensation, bone demineralization, loss of lamina dura, deep periodontal pockets, changes in saliva and poor oral hygiene are often associated with this condition, based on individual predisposition and the severity of the disease [1]. The knowledge of bone-related oral lesions in CRD patients is limited.

Cemento-ossifying fibroma (COF) is a distinct form of benign fibroosseous lesions of the mandible and maxilla containing fibrous tissue and varying amounts of calcified tissue resembling bone, cementum, or both [2]. The pathologic nature of COF is not yet clearly understood. It is included under neoplastic group of fibro osseous lesions thought to arise from periodontal ligament [2]. The aim of this report is therefore to present a case of COF who was also suffering from CRD and describe clinical characteristics of these conditions.

## 2. Case Report

A 37-year-old patient reported to outpatient department of our institution with a complaint of swelling of the lower jaw of six-month duration. There was neither a history of trauma or teeth extraction related to the swelling nor any similar swelling in other parts of the body. Patient's history revealed that she had been suffering from chronic renal Disease for the past six years. She had also been undergoing hemodialysis once a week for the past two years. On further questioning, patient revealed that she was suffering from pain in all joints. Patient was having hypertension secondary to the declining kidney function, for which she was under constant medication. The patient was manual labourer in farms for the past 20 years. Because of her declining physical condition she had stopped her work for the past four years.

**Figure 1:**
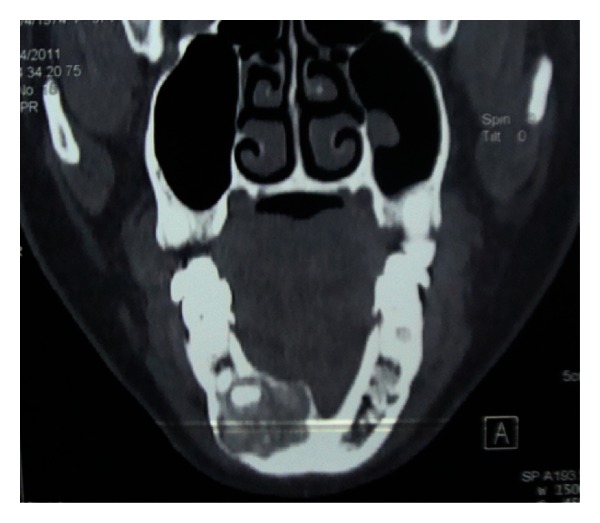
Axial CT image showing the lesion causing thinning and expansion of buccal as well as lingual cortical plate.

**Figure 2:**
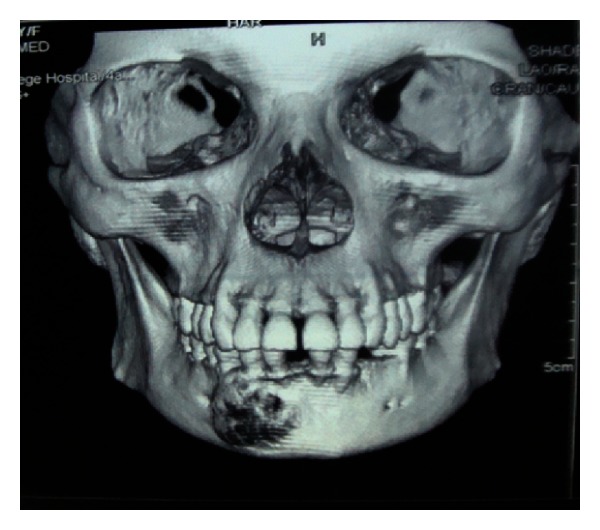
3D reformatted CT illustrating size and location of the well-defined tumor.

**Figure 3:**
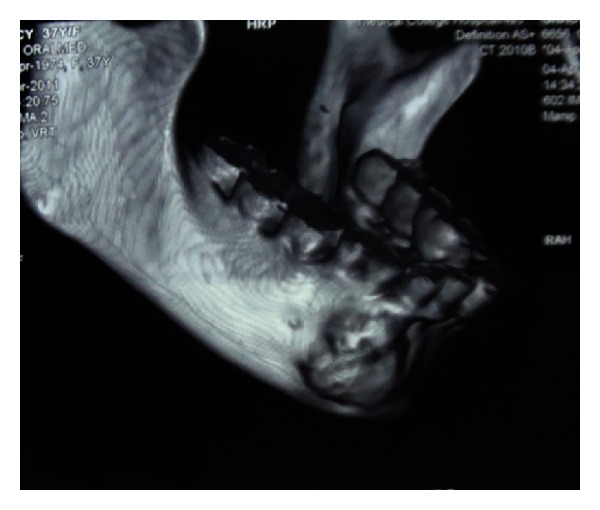
3D reformatted CT illustrating size and location of the well-defined tumor.

**Figure 4:**
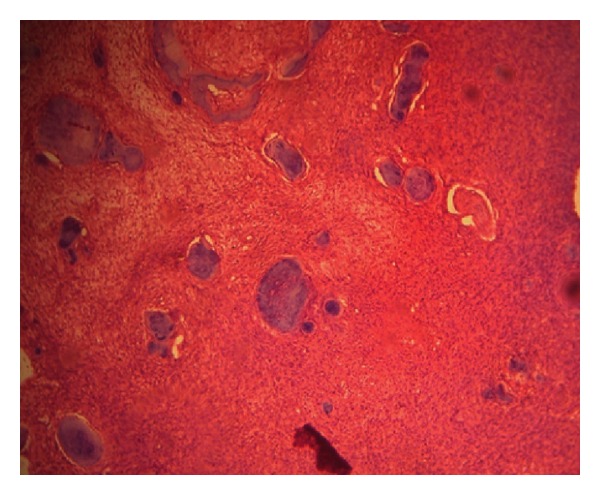
Fibro-cellular stroma containing cementum-like hard tissue calcifications (H&E stained section 10x)

Extra oral findings were nonremarkable. Intraoral examination revealed a nontender bony hard swelling of size 1.5 × 2 cm on the right mandibular body extending from central incisor to the first premolar with buccal cortical plate expansion. The overlying mucosa appeared to be normal. Pulp vitality of the teeth adjacent to the lesion proved positive. Orthopantomogram revealed an ill-defined radiolucency with radio-opaque specks located in the mandibular body. Root resorption was noted for the right canine and first premolar. Axial imaging showed the lesion to cause thinning and expansion of buccal as well as lingual cortical plate (Figure 1). Likewise, the core region of the lesion showed flecks of radiopacity. 3D reformatted computed tomography illustrated size and location of the well-defined tumor (Figures 2 and 3). A sample of the lesion was obtained for histopathological study, which revealed the presence of a fibrocellular stroma containing cementum-like hard tissue calcifications and other areas presenting recently formed osteoid with peripheral osteoblasts and signs of progressive calcification (Figure 4). There were no atypia or mitotic figures. The diagnosis was suggestive of cemento-ossifying fibroma. After medical clearance, surgical excision was carried out under local anaesthesia. The lesion was present as tiny irregular granulated fibrous tissue and pieces of bone-like immaturely calcified material. Teeth associated with the lesion were extracted, and curettage was performed to remove all fragments which were firmly attached to the underlying bone structure. The patient was clinically and radiographically symptom-free at the 18-month followup. Patient was advised orthopedic consultation for any other bony lesions, but the reports were inconclusive. But her renal condition was worse than before, and she was undergoing dialysis twice a week.

## 3. Discussion

The concept of “fibroosseous lesions” of bone evolved over the last several decades to include two major entities: fibrous dysplasia and ossifying fibroma as well as the other less common. It is a slow-growing lesion most often seen in women between the third and fourth decades of life. Both jawbones can be affected, although 62% to 89% of the lesions arise in the mandible, involving premolar and molar regions in 77% of cases. While one-half of all cases are asymptomatic, the growth of the tumor over time may lead to facial asymmetry, with the appearance of a mass causing discomfort or mandibular expansion and the possible displacement of dental roots.

Cemento-ossifying fibroma is a thought to have its origin from arises from the periodontal membrane [3]. The periodontal membrane is the layer of fibrous connective tissue surrounding the roots of teeth. It contains multipotent cells capable of forming cementum, lamellar bone, and fibrous tissue [4, 5]. However, lesions with similar histopathological features have been reported in other craniofacial structures and in long bones which do not contain cementum [6]. In addition, bone and cementum are both originated from mesenchymal stem cells. Accordingly, the classification of the World Health Organization suggested the term ossifying fibroma and considered it as a nonodontogenic neoplasm [7]. But cementum forming cells have been demonstrated to be phenotypically distinct from bone forming osteoblasts, and immunohistochemical staining differences have been reported with cementocytes being positive for fibromodulin and lumican whilst the bone forming cells are negative [8, 9]. Cementum lacks the lamellar architecture of mature bone and has been described as resembling a primitive, fetal type woven bone [10].

Cemento-ossifying fibroma is a slow growing lesion composed of cellular fibroblastic tissue containing basophilic masses of cementum-like tissue. In addition, a varying amount of bony trabeculae is interspersed within the lesion, giving it its characteristic appearance [11]. Reed used the presence or absence of woven and lamellar bone in histopathological section to differentiate the cement-ossifying fibroma from the other osseous lesions [12]. Fibrous dysplasia contains no lamellar bone but rather has a woven bone. On the other hand, cemento-ossifying fibroma and the ossifying fibroma contain woven bone that is often rimmed by osteoblasts that have laid down layers of lamellar bone [13]. Additionally, cemento-ossifying fibroma may have areas of cementum, appearing as psammoma bodies embedded in a benign fibrous stroma [13].

Radiological findings suggests that the lesion is usually well circumscribed and demarcated from surrounding bone, in contrast to true fibrous dysplasia. The central cementifying fibroma and the central ossifying fibroma, have a centrifugal growth pattern rather than a linear one. Therefore, lesions grow by expansion equally in all direction and present as round tumor masses.

The association of ossifying fibromas with renal diseases can be observed in hyperparathyroidism-jaw tumor syndrome, in which the patients suffer from familial parathyroid adenomas, ossifying fibroma of the jaws, renal cysts, and Wilms tumors [2, 14]. The HRPT2 gene has been found to be mutated in this syndrome, and it inactivates the parafibromin protein which has antiproliferative properties [14]. More recent studies have found perturbations in this gene among nonsyndrome associated ossifying fibromas of the jaws [15]. But only one case report describes an association of cementoossifying fibroma with end-stage renal disease [14]. The findings were similar to ours with normal PTH levels and no sign of parathyroid adenoma. Renal osteodystrophy, the term used to describe the skeletal complications of end-stage renal disease, is a multifactorial disorder of bone remodelling. Oral manifestations of the CRD due to renal osteodystrophy are late sign of renal disease and are usually due to alterations in calcium and phosphorum metabolism, abnormal metabolism of vitamin D, and the compensatory hyperactivity of parathyroid glands (secondary hyperparathyroidism). It is characterized by the following signs: bone demineralization, decreased trabeculation, decreased thickness of cortical bone, ground-glass appearance of bone, metastatic soft-tissue calcifications, radiolucent fibrocystic lesions, radiolucent giant cell lesions, lytic areas of bone, jaw fracture (spontaneous or after dental procedures), abnormal bone healing after extraction, and, sometimes, dental mobility as a consequence of the loss of substance in the bone [16]. But in the earlier stages of CRD, little is known about changes in bone metabolism and the effect of disturbed mineral metabolism on bone histology [17]. In the incipient stages of CRD, normal or mildly elevated PTH concentrations have until recently seldom resulted in therapy and thus may have mitigated the demand for bone biopsy; however, it has become evident that disturbances in bone metabolism present very early in CRD [17]. Humoral factors that are involved in the maintenance of normal bone homeostasis are usually perturbed as soon as the GFR (glomerular filtration rate) drops below 60 to 70 mL/min [18]. Serum FGF-23 (fibroblast growth factor) levels increase as soon as the GFR decreases below 60 mL/min, even before the development of hyperphosphatemia and hyperparathyroidism [19]. Moreover, additional studies have shown that the maximal tubular reabsorption of phosphate negatively correlates with serum concentrations of FGF-23 in stage 3 CRD, whereas in advanced CRD, this correlation is mitigated [19, 20]. Nevertheless, the increased FGF-23 further aggravates calcitriol deficiency by directly inhibiting the renal 1*α*-hydroxylase activity [20]. Both FGF-23 and calcitriol are important regulators of bone homeostasis [21]. Calcitriol and its receptor, the vitamin D receptor (VDR), are critical for normal bone metabolism, because appropriate activation of the VDR is necessary for normal osteoblastic bone formation and osteoclastic bone resorption, as well as for the coupling of osteoblastic and osteoclastic activity [21]. Studies have shown that FGF-23 overexpression is associated with severe skeletal phenotype characterized by disturbed mineralization process and growth plate architecture; however, they could not differentiate between local skeletal action and systemic effects of FGF-23 [22]. In addition, recently published data suggests that FGF-23 is a negative regulator of PTH mRNA expression and secretion in vitro as well [23]. Thus, a possible association of high FGF-23 with low PTH and calcitriol concentrations coupled with down regulation of the VDR may adversely affect bone metabolism, resulting in impaired bone turnover in the early CRD stages [17]. This speculation requires further validation. But whether there is a possible relation between FGF associated bone changes in CRD and the COF remains to be elucidated.

Due to the good delimitation of the COF, surgical removal and curettage is the treatment of choice. In the case of very large lesions with important tissue ablation, the challenge is to replace the affected tissue. The prognosis is usually good, since recurrences are not frequent.

## 4. Conclusion

Like any other systemic disease, CRD can also present with oral manifestations. Bony alterations in CRD are usually related to renal osteodystrophy. Further studies are needed to elucidate the pathogenesis of ossifying fibroma in patients with CRD.
